# Supplementary material for: Consequences of PCA graphs, SNP codings, and PCA variants for elucidating population structure
Source: PLoS One. 2019 Jun 18;14(6):e0218306. doi: 10.1371/journal.pone.0218306 (PMC6581268; doi:10.1371/journal.pone.0218306)
Supplement: S2 Table — (DOCX) [file pone.0218306.s002.docx]

**Table 1. Augmented ANOVA table for SNP-Centered PCA of oat data, using SNP coding VCF with oat line 189 as the reference genome.**

———————————————————————————————————————————————————————————

Source df SS Individuals SxI

———————————————————————————————————————————————————————————

Total 851534 175776.962

SNPs 1340 35206.937

I&SxI 850194 140570.025 6286.978 134283.047

PC1 1974 16325.612 5072.449 11253.163

PC2 1972 9751.158 279.884 9471.275

PC3 1970 6250.504 78.151 6172.353

PC4 1968 4860.910 503.165 4357.745

PC5 1966 3510.312 16.352 3493.959

PC6 1964 3181.953 45.154 3136.799

PC7 1962 2891.656 23.786 2867.870

Residual 836418 93797.920 268.037 93529.883

———————————————————————————————————————————————————————————

**Table 2. Augmented ANOVA table for SNP-Standardized PCA of oat data, using SNP coding rare=1.**

————————————————————————————————————————————————————————

Source df SS Individuals SxI

————————————————————————————————————————————————————————

I&SxI 850194 850194.000 14427.785 835766.215

PC1 1974 84215.540 5870.564 78344.976

PC2 1972 54117.580 1751.478 52366.102

PC3 1970 34779.168 3713.961 31065.207

PC4 1968 29068.093 279.528 28788.565

PC5 1966 23181.728 168.888 23012.840

PC6 1964 21843.243 0.403 21842.840

PC7 1962 17066.966 591.352 16475.614

Residual 836418 585921.681 2051.609 583870.072

————————————————————————————————————————————————————————

**Table 3. Augmented ANOVA table for Individual-Standardized PCA of oat data, using SNP coding rare=1.**

————————————————————————————————————————————————————————

Source df SS SNPs SxI

————————————————————————————————————————————————————————

S&SxI 850900 850900.000 93428.621 757471.379

PC1 1974 98971.642 81776.097 17195.545

PC2 1972 79236.782 11285.246 67951.536

PC3 1970 49788.726 2.118 49786.608

PC4 1968 33060.462 1.813 33058.649

PC5 1966 27932.581 215.481 27717.100

PC6 1964 19288.835 25.445 19263.390

PC7 1962 16246.322 5.008 16241.314

Residual 837124 526374.649 117.412 526257.237

————————————————————————————————————————————————————————

**Table 4. Augmented ANOVA table for Grand-Mean-Centered PCA of oat data, using SNP coding rare=1.**

—————————————————————————————————————————————————————————————————————

Source df SS Individuals SNPs SxI

—————————————————————————————————————————————————————————————————————

Total 851534 157442.756 2077.808 16872.731 138492.217

PC1 1975 17402.677 0.490 16741.733 660.454

PC2 1973 16325.068 859.333 7.902 15457.833

PC3 1971 9709.637 252.328 48.779 9408.530

PC4 1969 6217.325 441.158 16.201 5759.966

PC5 1967 4774.109 12.132 29.935 4732.042

PC6 1965 3490.992 11.347 4.086 3475.559

PC7 1963 3144.600 174.890 7.697 2962.013

Residual 837751 96378.348 326.130 16.398 96035.820

—————————————————————————————————————————————————————————————————————
