# Supplementary material for: Consequences of PCA graphs, SNP codings, and PCA variants for elucidating population structure
Source: PLoS One. 2019 Jun 18;14(6):e0218306. doi: 10.1371/journal.pone.0218306 (PMC6581268; doi:10.1371/journal.pone.0218306)
Supplement: S2 Fig — As in the main text, to reduce clutter, all of these biplots use two panels, with oat lines on the left and SNPs on the right. The color scheme is the same as in Fig 1 in the main text, namely spring oats show in green, world diversity oats shown in blue, and winter oats shown in red, with corresponding colors for the SNPs. (DOCX) [file pone.0218306.s004.docx]

These two figures show biplots for SNP-Centered and SNP-Standardized PCA for the oat data, using SNP coding rare=1. They are quite similar, and are nearly identical for both panels to Fig 1 in the main text, which uses DC-PCA and the same SNP coding.

**Fig 1. SNP-Centered PCA for the oat data, using SNP coding rare=1.**

**Fig 2. SNP-Standardized PCA for the oat data, using SNP coding rare=1.**

These two figures show biplots for Individual-Centered and Individual-Standardized PCA for the oat data, using SNP coding rare=1. They are similar, and are also similar for both panels to Fig 7 in the main text, which uses AMMI1 and the same SNP coding.

**Fig 3. Individual-Centered PCA for the oat data, using SNP coding rare=1.**

**Fig 4. Individual-Standardized PCA for the oat data, using SNP coding rare=1.**

This figure shows a biplot for Grand-Mean-Centered PCA for the oat data, using SNP coding rare=1. This biplot is similar for both panels to Fig 7 in the main text, which uses AMMI1 and the same SNP coding.

**Fig 5. Grand-Mean-Centered PCA for the oat data, using SNP coding rare=1.**
